# Supplementary material for: Aromatase inhibition and ketamine in rats: sex-differences in antidepressant-like efficacy
Source: Biol Sex Differ. 2023 Oct 24;14:73. doi: 10.1186/s13293-023-00560-5 (PMC10599051; doi:10.1186/s13293-023-00560-5)
Supplement: Supplementary file 1 — Additional file 1: Table S1. Antidepressant-like effects induced by ketamine or letrozole in adult male and female rats. Table S2. Antidepressant-like effects of ketamine in letrozole pre-treated male and female rats. [file 13293_2023_560_MOESM1_ESM.pdf]

Supplemental Tables

Supplemental Table S1. Antidepressant-like effects induced by ketamine or letrozole in adult male and female rats. This table represents the two-way ANOVAs analyses (F(DFn, DFd), *p* value for data represented in Fig. 2 (Study I) and 3 (Study II). Green-shadow boxes represent statistically significant comparisons.

**Study I: Sex differences in the antidepressant-like effects of ketamine**			
**Fig. 2A. 30 min FST**	**Sex**	**Treatment (Sal vs. Ket)**	**Sex x Treatment**
Immobility (s)	F(1,28) = 3.68; *p* = 0.065	F(1,28) = 0.34; *p* = 0.565	F(1,28) = 2.03; *p* = 0.165
Climbing (s)	F(1,28) = 5.19; #*p* = 0.031	F(1,28) = 0.01; *p* = 0.970	F(1,28) = 1.64; *p* = 0.211
Swimming (s)	F(1,28) = 0.15; *p* = 0.704	F(1,28) = 4.14; *p* = 0.052	F(1,28) = 1.53; *p* = 0.226
**Fig. 2B. 1 d FST**	**Sex**	**Treatment (Sal vs. Ket)**	**Sex x Treatment**
Immobility (s)	F(1,27) = 0.26; *p* = 0.614	F(1,27) = 6.34; *p* = 0.018	F(1,27) = 6.09; *p* = 0.020
Climbing (s)	F(1,27) = 0.22; *p* = 0.642	F(1,27) = 2.58; *p* = 0.120	F(1,27) = 5.58; *p* = 0.026
Swimming (s)	F(1,27) = 0.33; *p* = 0.569	F(1,27) = 5.77; *p* = 0.023	F(1,27) = 1.57; *p* = 0.221
**Fig. 2C. 3 d NSF**	**Sex**	**Treatment (Sal vs. Ket)**	**Sex x Treatment**
Feeding time (s)	F(1,28) = 0.30; *p* = 0.587	F(1,28) = 0.48; *p* = 0.492	F(1,28) = 0.03; *p* = 0.856
Distance (cm)	F(1,26) = 6.76; #*p* = 0.015	F(1,26) = 0.13; *p* = 0.720	F(1,26) = 0.37; *p* = 0.550
**Fig. 2D. 6-7 d SP**	**Sex**	**Treatment (Sal vs. Ket)**	**Sex x Treatment**
Intake (g/kg)	F(1,28) = 46.07; ###*p* < 0.001	F(1,28) = 0.20; *p* = 0.654	F(1,28) = 3.40; *p* = 0.076
Preference (%)	F(1,28) = 0.09; *p* = 0.767	F(1,28) = 1.55; *p* = 0.224	F(1,28) = 1.33; *p* = 0.259
**Study II: Sex differences in the antidepressant-like effects of letrozole**			
**Fig.3A. 1 h ELISA**	**Sex**	**Pre-treatment (Veh vs. LTZ)**	**Sex x Pre-treatment**
Testosterone (ng/ml)	F(1,20) = 10.81; ##*p* = 0.004	F(1,20) = 0.04; *p* = 0.835	F(1,20) = 1.43; *p* = 0.247
**Fig.3B. 1 h FST**	**Sex**	**Pre-treatment (Veh vs. LTZ)**	**Sex x Pre-treatment**
Immobility (s)	F(1,28) = 25.36; ###*p* < 0.001	F(1,28) = 0.06; *p* = 0.801	F(1,28) = 4.52; *p* = 0.043
Climbing (s)	F(1,28) = 21.28; ###*p* < 0.001	F(1,28) = 0.09; *p* = 0.763	F(1,28) = 1.69; *p* = 0.212
Swimming (s)	F(1,28) = 1.32; *p* = 0.261	F(1,28) = 0.06; *p* = 0.811	F(1,28) = 8.17; *p* = 0.008
**Fig. 3C. 1 d FST**	**Sex**	**Pre-treatment (Veh vs. LTZ)**	**Sex x Pre-treatment**
Immobility (s)	F(1,28) = 10.59; ##*p* = 0.003	F(1,28) = 0.58; *p* = 0.454	F(1,28) = 0.17; *p* = 0.682
Climbing (s)	F(1,28) = 12.82; ##*p* = 0.001	F(1,28) = 0.66; *p* = 0.425	F(1,28) = 0.09; *p* = 0.766
Swimming (s)	F(1,28) = 0.30; *p* = 0.590	F(1,28) = 0.01; *p* = 0.979	F(1,28) = 0.29; *p* = 0.595

Supplemental Table S2. Antidepressant-like effects of ketamine in letrozole pre-treated male and female rats. This table represents the three or two-way ANOVAs analyses (F(DFn, DFd), *p* value for data represented in Fig. 4 and 5 (Study III). Green-shadow boxes represent statistically significant comparisons.

**Study III: Antidepressant-like effects of ketamine in letrozole pre-treated male and female rats**			
**Fig. 4A. 30 min FST**	**Sex**	**Pre-treatment (Veh vs. LTZ)**	**Treatment (Sal vs. Ket)**
Immobility (s)	F(1,49) = 16.23; ###*p* < 0.001	F(1,49) = 9.57; *p* = 0.003	F(1,49) = 5.93; *p* = 0.019
Climbing (s)	F(1,49) = 17.06; ###*p* < 0.001	F(1,49) = 6.52; *p* = 0.014	F(1,49) = 3.19; *p* = 0.080
Swimming (s)	F(1,49) = 1.20; *p* = 0.279	F(1,49) = 11.09; *p* = 0.002	F(1,49) = 12.90; *p* < 0.001
**Fig. 4B. 1 d FST**	**Sex**	**Pre-treatment (Veh vs. LTZ)**	**Treatment (Sal vs. Ket)**
Immobility (s)	F(1,49) = 12.96; ###*p* < 0.001	F(1,49) = 1.02; *p* = 0.318	F(1,49) = 12.83; *p* < 0.001
Climbing (s)	F(1,49) = 11.52; ##*p* = 0.001	F(1,49) = 0.83; *p* = 0.366	F(1,49) = 10.25; p = 0.002
Swimming (s)	F(1,49) = 0.59; *p* = 0.447	F(1,49) = 0.15; *p* = 0.696	F(1,49) = 1.71; *p* = 0.197
**Fig. 4C. 3 d NSF**	**Sex**	**Pre-treatment (Veh vs. LTZ)**	**Treatment (Sal vs. Ket)**
Feeding time (s)	F(1,49) = 0.16; *p* = 0.688	F(1,49) = 0.52; *p* = 0.472	F(1,49) = 5.19; *p* = 0.027
Distance (cm)	F(1,47) = 10.08; ##*p* = 0.003	F(1,47) = 1.51; *p*= 0. 225	F(1,47) = 0.19; *p* = 0.665
**Fig. 4D. 6-7 d SP**	**Sex**	**Pre-treatment (Veh vs. LTZ)**	**Treatment (Sal vs. Ket)**
Intake (g/kg)	F(1,13) = 23.55; ###*p* < 0.001	F(1,13) = 1.9; *p* = 0.190	F(1,13) = 2.0; *p* = 0.182
Preference (%)	F(1,13) = 0.53: *p* = 0.480	F(1,13) = 0.60; *p* = 0.454	F(1,13) = 0.03; *p* = 0.869
**Fig. 5A. 65 d FST**	**Sex**	**Pre-treatment (Veh vs. LTZ)**	**Treatment (Sal vs. Ket)**
Immobility (s)	F(1,44) = 7.25; ##*p* = 0.010	F(1,44) = 4.07; *p* = 0.049	F(1,44) = 4.97; *p* = 0.031
Climbing (s)	F(1,44) = 6.76; #*p* = 0.013	F(1,44) = 3.70; *p* = 0.061	F(1,44) = 4.35; *p* = 0.043
Swimming (s)	F(1,44) = 1.11; *p* = 0.301	F(1,44) = 0.75; *p* = 0.392	F(1,44) = 1.18; *p* = 0.283
**Study III: Antidepressant-like effects of ketamine in letrozole pre-treated male rats**			
**Fig. 4A. 30 min FST**	**Pre-treatment (Veh vs. LTZ)**	**Treatment (Sal vs. Ket)**	**Pre-treatment x Treatment**
Immobility (s)	F (1,24) = 5.37; *p* = 0.029	F (1,24) = 12.06; *p* = 0.002	F(1,24) = 5.24; *p* = 0.031
Climbing (s)	F(1,24) = 4.42; *p* = 0.043	F(1,24) = 9.16; *p* = 0.006	F(1,24) = 3.43; *p* = 0.076
**Fig. 4B. 1 d FST**	**Pre-treatment (Veh vs. LTZ)**	**Treatment (Sal vs. Ket)**	**Pre-treatment x Treatment**
Immobility (s)	F (1,24) = 0.61; *p* = 0.441	F (1,24) = 21.71; *p* < 0.001	F(1,24) = 0.32; *p* = 0.574
Climbing (s)	F (1,24) = 0.56; *p* = 0.463	F (1,24) = 16.59; *p* < 0.001	F(1,24) = 0.78; *p* = 0.385
**Fig. 4C. 3 d NSF**	**Pre-treatment (Veh vs. LTZ)**	**Treatment (Sal vs. Ket)**	**Pre-treatment x Treatment**
Distance (cm)	F(1,22) = 1.70; *p* = 0.205	F(1,22) = 0.82; *p* = 0.374	F(1,22) = 0.09; *p* = 0.769
**Fig. 4D. 6-7 d SP**	**Pre-treatment (Veh vs. LTZ)**	**Treatment (Sal vs. Ket)**	**Pre-treatment x Treatment**
Intake (g/kg)	F(1,7) = 0.27; *p* = 0.621	F(1,7) = 0.00; *p* > 0.999	F(1,7) = 0.03; *p* = 0.864

**Fig. 5A. 65 d FST**	**Pre-treatment (Veh vs. LTZ)**	**Treatment (Sal vs. Ket)**	**Pre-treatment x Treatment**
Immobility (s)	F (1,24) = 0.19; *p* = 0.671	F (1,24) = 10.61; *p* = 0.003	F(1,24) = 0.01; *p* = 0.950
Climbing (s)	F (1,24) = 0.17; *p* = 0.678	F (1,24) = 10.24; *p* = 0.004	F(1,24) = 0.01; *p* = 0.927
**Study III: Antidepressant-like effects of ketamine in letrozole pre-treated female rats**			
**Fig. 4A. 30 min FST**	**Pre-treatment (Veh vs. LTZ)**	**Treatment (Sal vs. Ket)**	**Pre-treatment x Treatment**
Immobility (s)	F(1,25) = 4.16; *p* = 0.052	F(1,25) = 0.11; *p* = 0.743	F(1,25) = 2.44; *p* = 0.131
Climbing (s)	F(1,25) = 2.10; *p* = 0.159	F(1,25) = 0.75; *p* = 0.394	F(1,25) = 0.89; *p* = 0.355
**Fig. 4B. 1 d FST**	**Pre-treatment (Veh vs. LTZ)**	**Treatment (Sal vs. Ket)**	**Pre-treatment x Treatment**
Immobility (s)	F(1,25) = 8.37; *p* = 0.008	F(1,25) = 0.23; *p* = 0.638	F(1,25) = 0.40; *p* = 0.533
Climbing (s)	F(1,25) = 9.44; *p* = 0.005	F(1,25) = 0.46; *p* = 0.502	F(1,25) = 0.02; *p* = 0.903
**Fig. 4C. 3 d NSF**	**Pre-treatment (Veh vs. LTZ)**	**Treatment (Sal vs. Ket)**	**Pre-treatment x Treatment**
Distance (cm)	F(1,25) = 0.12; *p* = 0.730	F(1,25) = 0.16; *p* = 0.692	F(1,25) = 0.01; *p* = 0.989
**Fig. 4D. 6-7 d SP**	**Pre-treatment (Veh vs. LTZ)**	**Treatment (Sal vs. Ket)**	**Pre-treatment x Treatment**
Intake (g/kg)	F(1,6) = 1.56; *p* = 0.258	F(1,6) = 2.49; *p* = 0.166	F(1,6) = 0.02; *p* = 0.899
**Fig. 5A. 65 d FST**	**Pre-treatment (Veh vs. LTZ)**	**Treatment (Sal vs. Ket)**	**Pre-treatment x Treatment**
Immobility (s)	F(1,20) = 5.62; *p* = 0.028	F(1,20) = 0.01; *p* = 0.960	F(1,20) = 0.85; *p* = 0.367
Climbing (s)	F(1,20) = 4.962; *p* = 0.038	F(1,20) = 0.01; *p* = 0.951	F(1,20) = 0.78; *p* = 0.387
